# Investigating long-term quality of life and depressive symptoms following fracture-related infection: a systematic review with meta-analysis of heterogeneous evidence

**DOI:** 10.5194/jbji-11-569-2026

**Published:** 2026-09-14

**Authors:** Johann Alexander Betker, Nike Walter, Markus Rupp, Susanne Baertl, Volker Alt

**Affiliations:** 1 Department for Trauma Surgery, University Hospital Regensburg, Franz-Josef-Strauß-Allee 11, 93053 Regensburg, Germany; 2 Department for Psychosomatic Medicine, University Hospital Regensburg, Franz-Josef-Strauß-Allee 11, 93053 Regensburg, Germany; 3 Department of Trauma, Hand and Reconstructive Surgery, University Hospital Gießen, Rudolf-Buchheim-Straße 7, 35392 Gießen, Germany

## Abstract

**Background**: Fracture-related infection (FRI) is associated with prolonged treatment courses and functional impairment. However, the long-term effects of FRI on quality of life (QoL) and psychological outcomes remain unclear.

**Methods**: A PRISMA-compliant systematic review of PubMed, Web of Science, and Google Scholar for studies published from 2010 to August 2025 was performed. Methodological quality and bias were assessed with Methodological Index for Non-Randomised Studies (MINORS) and ROBINS-I. Where appropriate, data were synthesized using random-effects models and interpreted cautiously due to substantial heterogeneity.

**Results**: Fifteen studies met the inclusion criteria. The evidence base is dominated by retrospective cohorts: mean MINORS attainment 66.4 % and mean loss to follow-up 19.5 %. Meta-analysis of three studies ( n=125 ) showed non-significant mean changes at 12 months or more in SF-36 and SF-12 physical component score (PCS) of 5.81 (SD 6.54, p=0.124 ) and 3.86, (9.12, p=0.463 ) in the mental component (MCS). Pooled long-term estimates from seven studies ( n=262 ) were 38.9 (2.18) for PCS and 51.2 (4.11) for MCS. Compared with US population norms, PCS was reduced, whereas differences in mental QoL were not significant. Three studies ( n=118 ) assessing psychological symptoms using the ISR reported elevated depressive symptom and total ISR scores exceeding established caseness thresholds.

**Discussion**: Current evidence suggests persistent physical and psychological burden after FRI treatment. However, heterogeneity, limited study quality, evolving FRI definitions, and a lack of appropriate comparison groups prevent definitive conclusions. Prospective studies using standardized outcome measures are required.

## 1 Introduction

Fracture-related infections (FRIs) are widely regarded as a consequential and therapeutically challenging complication in orthopaedic and trauma surgery. Incidence is estimated at 1 %–2 % in closed fractures, yet rates may exceed 30 % in Gustilo–Anderson type III open fractures, depending on anatomical site and injury severity (Ktistakis et al., 2014; Metsemakers et al., 2017; Singh et al., 2012; Walter et al., 2025). The impact on patients is substantial. FRI induces persistent pain and functional limitation, while extending to considerable social and emotional sequelae. Traumatic injury alone may provoke adverse psychological outcomes (Vranceanu et al., 2014; Walter and Alt, 2025), but the superimposition of bone infection amplifies these effects through prolonged immobility, treatment uncertainty, and financially destabilizing work incapacity (Abulaiti et al., 2017; O'Hara et al., 2021; Walter et al., 2024). Therapeutic objectives aim simultaneously at infection eradication and restoration of biomechanical integrity, frequently necessitating meticulously staged, complex surgical protocols and protracted hospitalization. International consensus on a standardized definition of FRI has emerged only recently, and reported eradication rates range widely from 29 % to 87 % (Bezstarosti et al., 2019; Buijs et al., 2022; Lu et al., 2022; Walter et al., 2024). Therapeutic failure or persistent infection may result in irreversible functional compromise or amputation (Lu et al., 2022).

Infection recurrence portends intensified morbidity, extended therapy, cumulative psychological burden, and profound deterioration in quality of life (QoL) (Wimalan et al., 2023).

This systematic review synthesizes longitudinal evidence on QoL trajectories following revision surgery for FRI, with particular focus on psychological symptom burden. It addresses three central questions. First, do patients exhibit sustained reductions in quality of life metrics for 12 months or longer after treatment? Second, what physical and psychological outcomes can be reasonably anticipated 1 year or more following successful infection eradication? Third, are FRI patients at demonstrably increased risk of developing depressive symptoms within the first postoperative year?

## 2 Methods

### 2.1 Data collection

A comprehensive, computerized, systematic search of PubMed, Web of Science, and Google Scholar was conducted to identify studies published from 2010 to August 2025. The review followed the Preferred Reporting Items for Systematic Reviews and Meta-Analyses (PRISMA) guidelines (Moher et al., 2010).

Given the heterogeneous terminology in the literature – including osteomyelitis, fracture-related infection, and infected non-union (Rupp et al., 2021) – the search strategy encompassed all relevant descriptors combined with “quality of life”, “mental health”, “depression”, “pain”, “mobility”, “anxiety”, and “patient-reported outcome measures”.

Two independent reviewers screened all citations and manually examined reference lists to ensure completeness. Eligible studies included adult patients with FRI, excluded vertebral cases, had more than five participants, were published in English, employed case-control or cohort designs, and assessed quality of life with validated instruments at a minimum follow-up of 12 months. Levels of evidence (LoE) were assigned according to the Oxford Centre for Evidence-Based Medicine (OCEBM) framework, with ambidirectional cohorts classified as retrospective for consistency (Howick et al., 2025; Marx et al., 2015; Wright et al., 2003).

Methodological quality was independently appraised using the Methodological Index for Non-Randomised Studies (MINORS) checklist (Slim et al., 2003), and discrepancies were resolved by consensus meetings. Risk of bias (RoB) was assessed via the Risk Of Bias In Non-Randomised Studies – of Interventions (ROBINS-I) framework, assigning low, moderate, or high risk across seven domains (Sterne et al., 2016; Sterne et al., 2019).

### 2.2 Statistical analysis

Data were classified into three groups: studies evaluating pre- and postoperative QoL within the same cohort, studies comparing postoperative outcomes between cohorts, and studies assessing the risk of depressive symptoms following fracture-related infection. Due to considerable variation in patient-reported outcome measures (PROMs), instruments were examined for equivalence, and data from related instruments measuring comparable domains were pooled. Postoperative outcomes were analysed at 12 months or longer following surgery.

Weighted mean differences with standard deviations and 95 % confidence intervals relative to baseline were calculated, with normality assessed using the Shapiro–Wilk test and changes evaluated via z tests. Robustness was confirmed through leave-one-out sensitivity analyses.

Physical and mental domains of PROMs were evaluated separately. A random-effects model accounted for inter-study variability. Statistical heterogeneity was assessed using Cochran's Q test and the $I^{2}$ statistic, complemented by visual inspection of forest plots for discrepancies. Publication bias was evaluated with Egger's test and a visual examination of funnel plots, with p values above 0.05 indicating the absence of bias. When bias was detected, sensitivity analyses were conducted. A two-sided p value was used for comparison of the pooled estimates with US normative values.

Psychological impact was quantified using weighted means across different syndromes, with corresponding standard deviations and 95 % CIs, and robustness was verified through sensitivity analysis. All analyses and visualizations were performed in R version 4.4.1 (R Core Team, 2024).

## 3 Results

### 3.1 Data extraction

**Figure 1:**
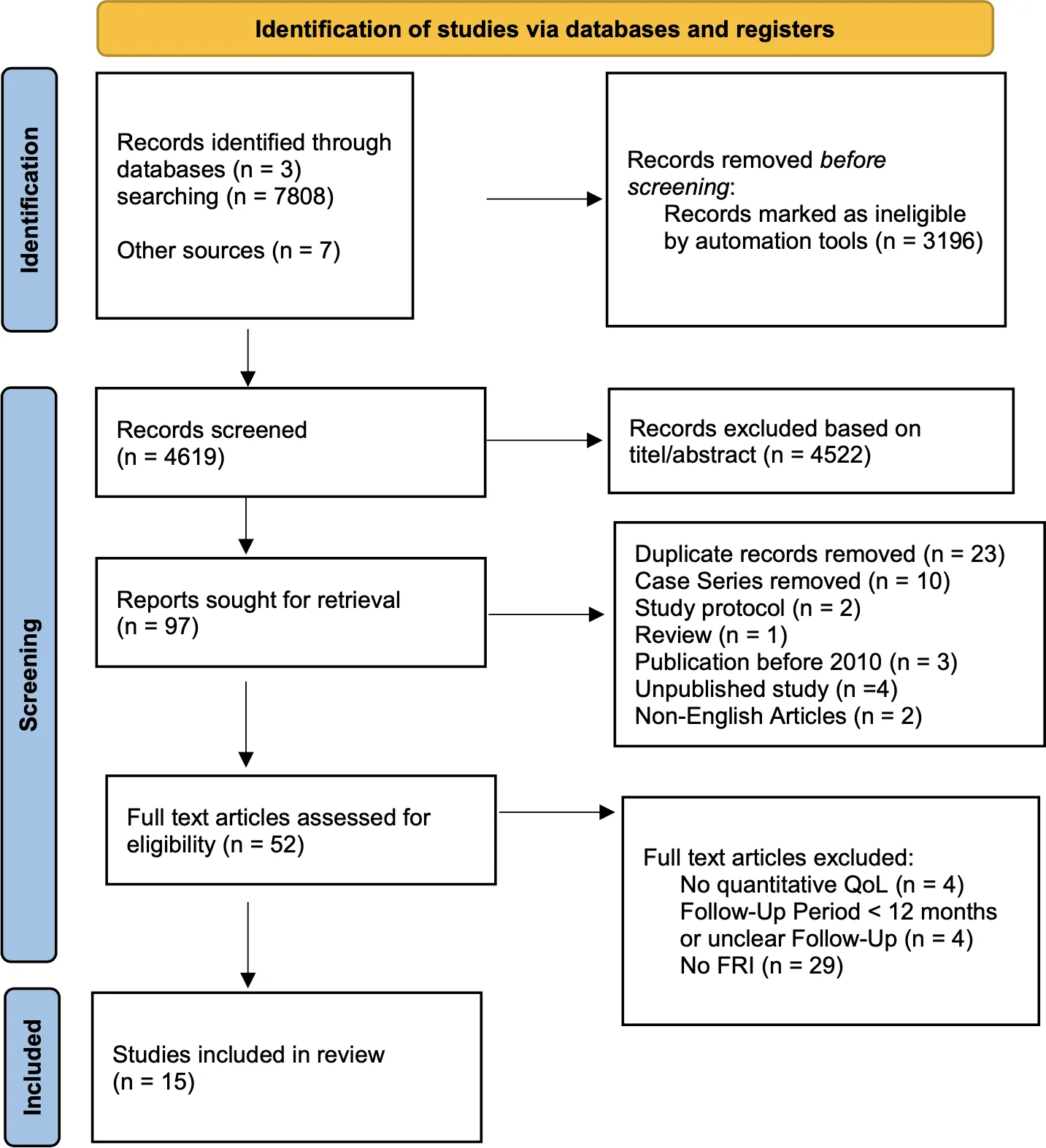
Selection process of eligible studies according to the PRISMA guidelines.

The systematic search across PubMed, Web of Science, and Google Scholar identified 7808 articles, with an additional seven records retrieved from references in relevant reviews. Automated database filters excluded 3196 records prior to screening. Of the remaining 4619 articles, 4522 were discarded following title and abstract review. Duplicates, inadvertently included case reports, study protocols, studies published before 2010, a review, unpublished studies limited to abstracts, and non-English studies were subsequently removed. Following comprehensive abstract evaluation and reference list screening, 52 full-text articles were selected for detailed assessment, as summarized in Fig. 1.

Studies lacking standardized QoL or mental health assessments, those with inadequate follow-up, and studies without cohorts exclusively diagnosed with FRI were excluded. Fifteen studies ultimately met the inclusion criteria (Buijs et al., 2024; Capdevilla et al., 2024; Egeler et al., 2018; Iliaens et al., 2021; Koutalos et al., 2021; Maurer et al., 2022; Raven et al., 2019; Rodham et al., 2023; Walter et al., 2021, 2024, 2025; Wang et al., 2017; Yilihamu et al., 2017; Zayzan et al., 2022; Zhang et al., 2023).

### 3.2 Methodological quality

Among the included studies, two were classified as level II evidence, eight as level III, and five as level IV, indicating a moderate to weak overall quality of evidence. No study achieved level I evidence (Table 1).

Risk of bias assessment revealed that 10 studies exhibited moderate risk, two studies were rated as low (Raven et al., 2019; Walter et al., 2025), and three as high risk (Buijs et al., 2024; Capdevilla et al., 2024; Koutalos et al., 2021). The primary source of bias was within the first domain ( n=15 ). A substantial portion of these studies were retrospective in design and did not fully account for potential confounders. Only two studies blinded the evaluation of endpoints (Buijs et al., 2024; Zayzan et al., 2022).

MINORS scores indicated moderate to weak methodological quality, with studies achieving a mean of 66.39 % of the maximum score (range 50.00 %–91.67 %). Average loss to follow-up was 19.54 %, ranging from 0.00 % to 57.32 % (Table 1).

**Table 1:** Level of evidence and MINORS score for each included study.

Author (year)	Study type	Level of evidence	1	2	3	4	5	6	7	8	9	10	11	12	Σ
Buijs et al. (2024)	Retrospective case-control study	III	2	2	1	2	1	2	1	0	2	2	1	2	18
Capdevilla et al. (2024)	Retrospective cohort study	III	2	2	0	2	0	2	2	0	2	0	1	2	15
Egeler et al. (2018)	Retrospective cohort study	III	2	2	0	2	0	2	0	0	2	2	0	1	13
Iliaens et al. (2021)	Retrospective case-control study	III	2	2	0	2	0	2	0	0	2	2	1	1	14
Koutalos et al. (2021)	Retrospective case-control study	III	2	2	0	2	0	2	0	0	2	1	1	1	13
Maurer et al. (2022)	Retrospective single-arm cohort study	IV	2	2	0	2	0	2	1	0	–	–	–	–	9
Raven et al. (2019)	Prospective cohort study	II	2	2	2	2	0	2	2	2	2	2	2	2	22
Rodham et al. (2023)	Retrospective single-arm cohort study	IV	2	2	2	2	0	2	1	0	–	–	–	–	11
Walter et al. (2021)	Retrospective cohort study	III	2	2	0	2	0	2	0	0	–	–	–	–	8
Walter et al. (2024)	Prospective cohort study	II	2	2	2	2	0	2	2	0	2	2	1	1	18
Walter et al. (2025)	Prospective single-arm cohort study	IV	2	2	2	2	0	2	2	0	–	–	–	–	12
Wang et al. (2017)	Retrospective single-arm cohort study	IV	2	2	2	2	0	2	2	0	–	–	–	–	12
Yilihamu et al. (2017)	Retrospective cohort study	III	2	1	0	2	0	2	2	0	1	0	2	0	12
Zayzan et al. (2022)	Retrospective single-arm cohort study	IV	2	1	0	2	2	2	1	2	–	–	–	–	12
Zhang et al. (2023)	Retrospective cohort study	III	2	2	0	2	0	2	2	0	2	2	2	1	17

### 3.3 QoL development after surgery

Four studies were initially considered (Capdevilla et al., 2024; Raven et al., 2019; Walter et al., 2025; and Wang et al., 2017), with one excluded for not employing standardized RAND Corporation instruments (Capdevilla et al., 2024).

**Figure 2:**
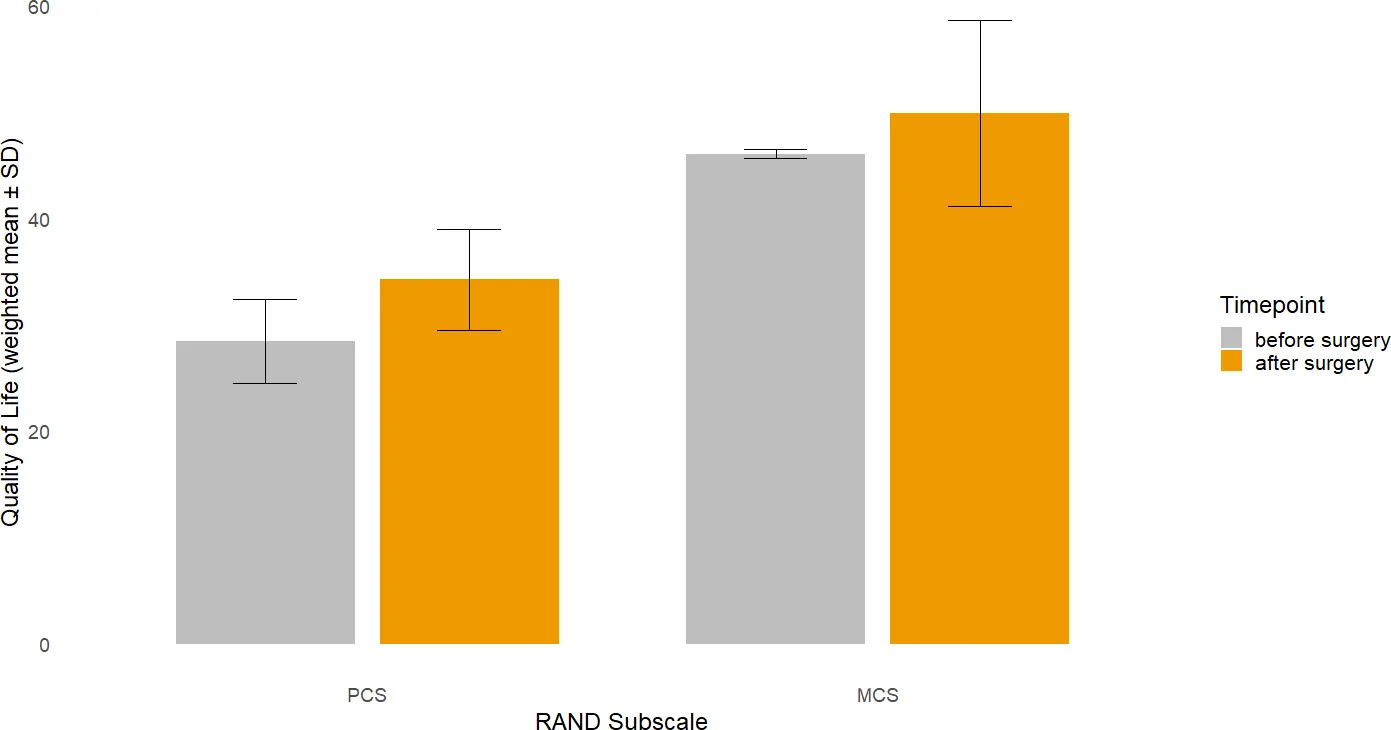
Weighted mean physical component score and mental component score, and standard deviations before and 12 months or more after surgery.

Meta-analysis of 125 patients from three studies demonstrated an average increase in physical component summary (PCS) at 12 months or longer of 5.81 (SD 6.54; 95 % CI [ -1.59 ; 13.21]), which did not reach statistical significance ( z=1.54 , p=0.124 ). The mental component score (MCS) increased by 3.86 points on average (9.12; [ -6.46 ; 14.18]), also non-significant ( z=0.733 , p=0.463 ). Trajectories are depicted in Fig. 2.

Excluding a single study produced only small changes in the statistical significance of the weighted mean difference in PCS (range of change: Δ-2.41 to 2.27; |z|<0.4 ), whereas the statistical significance of the weighted mean difference in MCS was moderately affected ( Δ-3.18 to 5.11; |z|<0.6 ).

### 3.4 Long-term effects on QoL

Seven studies, comprising 262 patients, provided comparable long-term QoL data using RAND Corporation instruments (Egeler et al., 2018; Maurer et al., 2022; Raven et al., 2019; Walter et al., 2021, 2025; Wang et al., 2017; Zayzan et al., 2022).

Random-effects meta-analysis revealed a pooled PCS estimate of 38.9 (SD = 2.18; 95 % CI [34.6–43.2]). Between-study heterogeneity was substantial ( $\tau^{2}=27.3$ ; $I^{2}\approx 85.7$ %), although the Egger regression test indicated no evidence of publication bias ( z=1.07 , p=0.29 ). In comparison with normative data (mean = 49.22, SD = 15.13, n=3844 ) from the US population (Maglinte et al., 2012), FRI patients demonstrated a significantly lower PCS (SE = 2.20; z=-4.69 ; p<0.001 ).

For the MCS, the pooled estimate was 51.2 (4.11; [43.1; 59.3]), with extreme heterogeneity across studies ( $\tau^{2}=112.9$ ; $I^{2}\approx 97.8$ %). Again, there was no indication of significant funnel plot asymmetry (Egger z=-0.15 , p=0.88 ). Pooled MCS showed no significant impairment (SE = 4.12; z=-0.63 ; p=0.53 ) in mental-health-related QoL when compared to US population norm (mean = 53.78, SD = 13.14, n=3844 ) (Maglinte et al., 2012).

**Figure 3:**
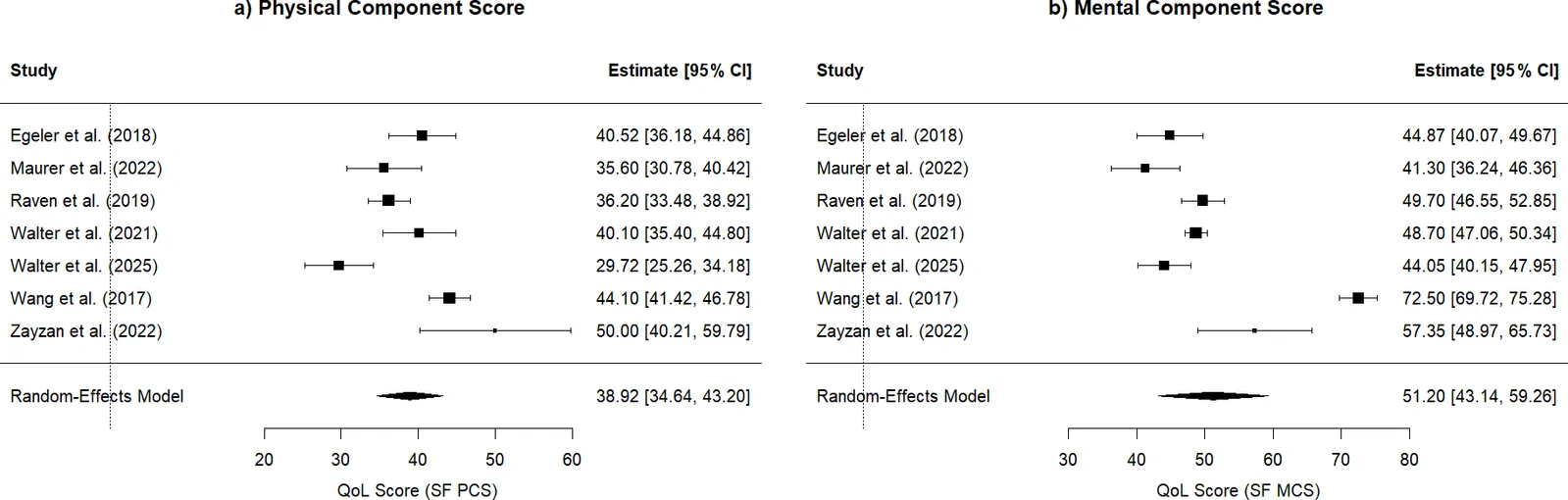
Forest plot depicting the estimated effect, effect size, and confidence interval for **(a)** physical component score (PCS) and **(b)** mental component score (MCS) based on SF-36 and SF-12 for each included study.

The pronounced heterogeneity, particularly for MCS, suggests that study-specific, clinical, and methodological factors strongly influence observed outcomes. Therefore, pooled estimates should be interpreted as reference benchmarks rather than precise predictions for individual studies. Forest plots are presented in Fig. 3.

### 3.5 Psychological burden

Three studies evaluated psychological outcomes 1 year or longer postoperatively using the ISR in 118 patients (Maurer et al., 2022; Walter et al., 2021, 2024).

**Figure 4:**
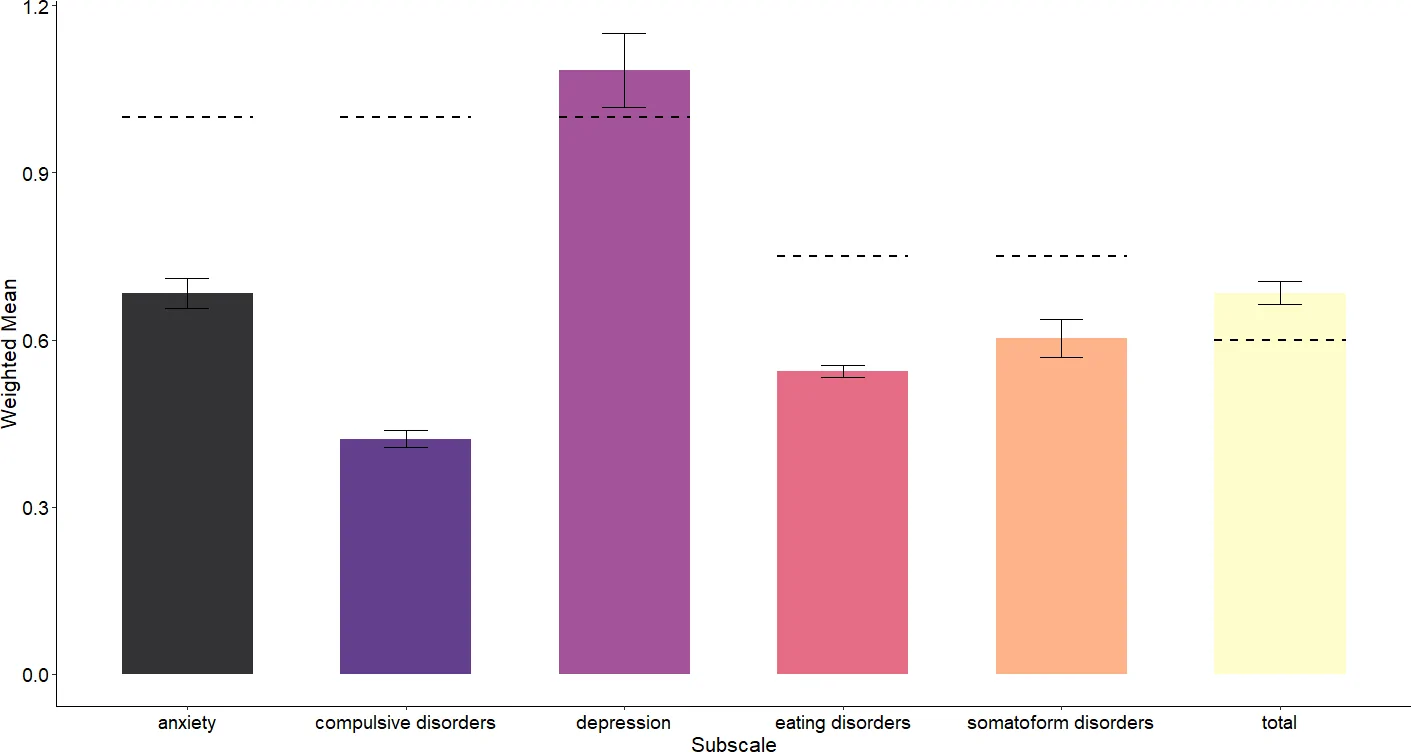
Weighted mean ICD-10-based symptom rating scores with 95 % CI and cut-off values for caseness.

At 12 months and more, the weighted mean psychological burden was 0.68 (SD 0.15; 95 % CI [0.66; 0.71]) for anxiety, 0.42 (0.06; [0.41; 0.44]) for compulsive disorder, 1.08 (0.37; [1.02; 1.15]) for depression, 0.54 (0.04; [0.53; 0.55]) for eating disorders, and 0.60 (0.14; [0.57; 0.64]) for somatoform disorders. The weighted mean total ISR score was 0.68 (0.11; [0.66; 0.70]). None of the weighted mean sub-scores in the syndrome scales met the criteria of caseness, except for the depression score crossing the cut-off value for mild psychological symptom burden (Tritt et al., 2008). Also, the ISR total score surpassed the cut-off values of caseness (Fig. 4).

A leave-one-out influence analysis showed that removing one study led to only small changes in the weighted mean for depression (range of change: Δ-0.12 to 0.14; |z|<0.4 ), and small to moderate changes in anxiety ( Δ-0.05 to 0.09; |z|<0.6 ) and the ISR total score ( Δ-0.06 to 0.06; |z|<0.55 ). Thus, caseness of depressive symptoms seems to be robust.

## 4 Discussion

This systematic review synthesized available evidence on long-term quality of life and psychological outcomes following fracture-related infection. Across 15 included studies, the findings suggest that patients treated for FRI may experience persistent impairments, particularly in physical-health-related quality of life, while psychological outcomes remain insufficiently characterized and yielded inconsistent results across different assessment instruments. However, interpretation of the available evidence requires considerable caution because the included studies demonstrated substantial clinical, methodological, and statistical heterogeneity.

The evidence base consisted predominantly of retrospective, unblinded cohort studies providing level III evidence. Overall methodological quality was moderate, reflected by a mean MINORS score of 66.4 %. According to the ROBINS-I assessment, confounding represented the principal source of bias. Furthermore, the mean loss to follow-up of approximately 20 % introduces uncertainty about the representativeness of long-term outcome estimates. These limitations substantially restrict the extent to which pooled estimates can be interpreted as reliable measures of the true long-term burden of FRI.

Only three studies examined changes in quality of life within the same cohort before and after treatment. The pooled analysis indicated numerical improvement in both the physical and mental component scores, yet neither reached statistical significance. The overall trend was largely driven by two studies reporting improved outcomes after treatment (Raven et al., 2019; Wang et al., 2017). In contrast, the largest cohort demonstrated a decline in QoL following surgery (Walter et al., 2025). This discrepancy underscores an important clinical reality: the successful eradication of infection does not inevitably restore patient-reported health status.

The pooled analysis of seven studies assessing long-term QoL between cohorts yielded mean PCS and MCS values of 38.9 and 51.2, respectively. Physical QoL was consistently lower than values reported for reference populations, whereas mental health appeared largely preserved. Nevertheless, these comparisons require careful interpretation. FRI populations are not necessarily representative of the general population, as patients may differ in age distribution, socioeconomic background, comorbidity burden, injury severity, and functional demands. Moreover, the included studies varied substantially in sample size and demographic composition, particularly with respect to age. Consequently, identifying an appropriately matched normative population proved difficult and limited the interpretability of these comparisons. Most cohorts also originated from German healthcare systems, whereas normative data were primarily derived from the US. Although previous work has shown broad agreement between German and US SF-36 reference values (Ellert and Kurth, 2004), regional variation in health-related QoL may still influence the observed differences.

Psychological health emerged as an important yet understudied aspect of recovery. Three studies comprising 118 patients reported depressive symptom scores above established ISR caseness thresholds, suggesting that psychological distress may persist after treatment. However, these results should not be interpreted as evidence of increased depression risk attributable to FRI, as the meta-analysis did not compare the FRI groups to an appropriately matched control group consisting of patients with comparable fractures, trauma exposure, or chronic conditions. Consequently, it remains unclear whether the observed symptoms reflect the effects of infection itself or the broader consequences of severe musculoskeletal injury and prolonged treatment. Future investigations should include appropriate comparator groups to disentangle these effects.

A major challenge of this review was the considerable heterogeneity between included studies. Variation existed in patient characteristics, demographics, fracture location, surgical strategies, treatment pathways, outcome instruments, and follow-up duration. The studies also spanned more than 15 years, during which the conceptual understanding and diagnostic framework of FRI evolved considerably. Although contemporary consensus definitions have improved diagnostic consistency (Walter et al., 2024), earlier investigations often relied on less-standardized criteria, introducing further variability into the evidence base.

This heterogeneity also influenced the interpretation of the meta-analysis. Random-effects models, sensitivity analyses, and assessments of publication bias were performed to address between-study variation. Nevertheless, the persistently high $\tau^{2}$ and $I^{2}$ values indicate considerable residual heterogeneity. The pooled estimates should therefore be viewed as descriptive summaries rather than universally applicable effect estimates. Statistical certainty is further constrained by the limited number of studies contributing to each analysis and z test results obtained from a small set of eligible studies carry substantial uncertainty (Röver et al., 2015). Consequently, hypothesis testing based on these datasets warrants cautious interpretation.

An alternative strategy would have been to conduct three separate systematic reviews addressing longitudinal changes in quality of life, cross-sectional comparisons between cohorts, and psychological outcomes after FRI. Such an approach, however, would have fragmented the already limited evidence base and substantially reduced the interpretability of each review. We therefore chose to integrate these related questions within a single systematic review. This strategy provides a comprehensive overview of the current literature while explicitly acknowledging the limitations imposed by the available evidence.

## 5 Conclusion

Future research should prioritize prospective, properly blinded studies using standardized FRI definitions, validated QoL instruments, longitudinal follow-up, and integrated psychological assessment. Carefully selected control groups are equally essential. Such studies would clarify whether the observed impairments arise primarily from infection, severe trauma, surgical treatment, or the cumulative burden of recovery.

## Data Availability

The dataset used for the meta-analysis as well as the R code can be made available upon reasonable request to the corresponding author.
